# Successful post-exposure prophylaxis of Ebola infected non-human primates using Ebola glycoprotein-specific equine IgG

**DOI:** 10.1038/srep41537

**Published:** 2017-02-03

**Authors:** Oleg V. Pyankov, Yin Xiang Setoh, Sergey A. Bodnev, Judith H. Edmonds, Olga G. Pyankova, Stepan A. Pyankov, Gabor Pali, Shane Belford, Louis Lu, Mylinh La, George Lovrecz, Valentina A. Volchkova, Keith J. Chappell, Daniel Watterson, Glenn Marsh, Paul R. Young, Alexander A. Agafonov, Jillann F. Farmer, Victor E. Volchkov, Andreas Suhrbier, Alexander A. Khromykh

**Affiliations:** 1 State Center for Virology and Biotechnology Vector, Koltsovo, Russian Federation; 2 Australian Infectious Diseases Research Centre, School of Chemistry and Molecular Biosciences, University of Queensland, St Lucia, Brisbane, QLD, Australia; 3 Plasvacc Pty. Ltd., Kalbar, QLD, Australia; 4 Bio Medical Manufacturing, Fermentation and Protein Production Facility, CSIRO, Clayton, VIC, Australia; 5 Molecular Basis of Viral Pathogenicity, CIRI, INSERM, U1111-CNRS UMR5308, Université de Lyon, Université Claude Bernard Lyon 1, Ecole Normale Supérieure de Lyon, France; 6 Australian Animal Health Laboratory, CSIRO Health and Biosecurity, Geelong, VIC, Australia; 7 United Nations Medical Service, New York, NY 10017, USA; 8 QIMR Berghofer Medical Research Institute, Brisbane, QLD, Australia

## Abstract

Herein we describe production of purified equine IgG obtained from horses immunized with plasmid DNA followed by boosting with Kunjin replicon virus-like particles both encoding a modified Ebola glycoprotein. Administration of the equine IgG over 5 days to cynomolgus macaques infected 24 hours previously with a lethal dose of Ebola virus suppressed viral loads by more than 5 logs and protected animals from mortality. Animals generated their own Ebola glycoprotein-specific IgG responses 9–15 days after infection, with circulating virus undetectable by day 15–17. Such equine IgG may find utility as a post-exposure prophylactic for Ebola infection and provides a low cost, scalable alternative to monoclonal antibodies, with extensive human safety data and WHO-standardized international manufacturing capability available in both high and low income countries.

The largest outbreak of Ebola virus (EBOV) ever recorded occurred in 2014–2016 primarily in West Africa and resulted in almost 30,000 infections and over 11,000 deaths, with exported cases in Europe and North America. The outbreak resulted in the establishment of the United Nations Mission for Ebola Emergency Response (UNMEER), only the second time that the Security Council has dealt directly with a public health problem. Accelerated vaccine development resulted in phase II human trials, and new therapies were actively pursued^1^. Amongst these was the development of monoclonal antibodies directed at the EBOV surface glycoprotein (GP) that were capable of neutralizing EBOV^2^,^3^. Post-exposure treatments with several such antibodies or antibody cocktails were capable of protecting non-human primates (NHPs) from fatal infections^4^,^5^,^6^. However, monoclonal antibodies require considerable investment for scale-up and manufacture and are expensive^7^. Antibodies from vaccinated horses provide a low-cost alternative, which were traditionally used to treat several diseases^8^, and remain in use for rabies, botulism and diphtheria (of Iditarod fame). Importantly, equine IgG products are widely available for treating envenomation and are manufactured in both high and low income countries, with the WHO providing standardized guidelines for production^9^.

In early studies, anti-EBOV IgG was generated by immunizing horses with culture fluid of EBOV-infected cells or with liver homogenates from infected guinea pigs; however, such equine IgG was only able to protect 100% of NHPs when high doses of IgG were administered before or within 1 hour of challenge with low doses of EBOV^10^,^11^. The immunogens used in these early studies were likely suboptimal as they would have contained large amounts of the secreted non-structural glycoprotein (sGP), which diverts the immune response into production of non-protective antibodies (a process known as “antigenic subversion”)^12^,^13^. Subsequent recombinant GP immunogen design (including that used herein) avoided sGP production by using an RNA edited version of GP^14^, which results in production of full length GP. More recent IgG studies have shown post-exposure protection in guinea pigs (using challenge with guinea pig-adapted EBOV) using (i) IgG from horses and pigs immunized with EBOV virus-like particles^15^,^16^, and (ii) serum from sheep immunized with recombinant GP^17^. However, these IgG products have not, to our knowledge, proceeded to NHP studies.

We have previously demonstrated high immunogenicity of Kunjin virus (KUN) replicon-based vectors in horses^18^ and have shown protective efficacy of a KUN replicon virus-like particle (VLP)-based vaccine (KUN-VLP-GP/D637L) against lethal EBOV challenge in guinea pigs^19^ and NHPs^20^. The KUN replicon VLPs encoded GP with a D637L mutation, which enhances (from a low baseline) GP cleavage on the cell surface by tumor necrosis factor-alpha converting enzyme (TACE) resulting in increased production of soluble shed GP^21^. The use of the D637L mutant results in presentation to the immune system of both the full-length membrane-bound GP and the soluble shed GP. Shed GP has been implicated in depletion of virus-neutralizing antibodies and pathogenicity during EBOV infection^21^,^22^. Our vaccine studies in guinea pigs showed that KUN replicon VLP vaccine producing the D637L mutant of GP provided better protection than VLPs producing wild-type GP at lower vaccine doses^19^. Herein we describe generation of anti-EBOV IgG in horses by immunization against GP/D637L using priming with a DNA vaccine (phCMV-GP/D637L), followed by a boost with a KUN replicon VLP vaccine (KUN-VLP-GP/D637L)^19^,^20^. We further show that post exposure treatment with this equine IgG protects NHPs from lethal EBOV infection.

## Results

### Generation and purification of anti-EBOV equine IgG

**Figure 1:**
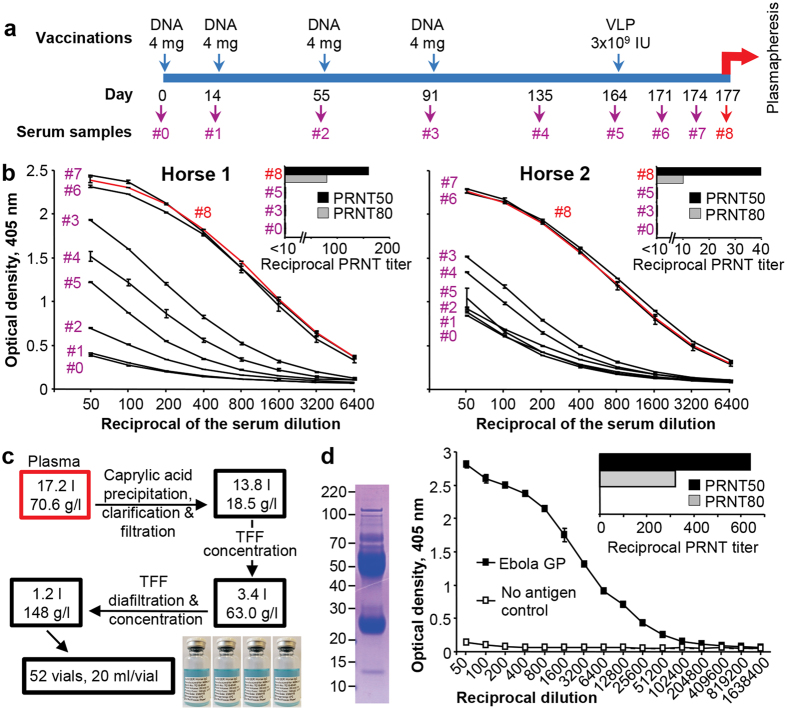
Production and purification of anti-EBOV GP equine IgG. (**a**) Schedule for horse immunization, serum sample collection and plasmapheresis. DNA - phCMV-GP/D637L. VLP - KUN-VLP-GP/D637L. (**b**) Anti-EBOV antibody titers of equine serum samples determined by ELISA. Inserts show virus neutralization titers; PRNT - plaque reduction neutralizing titers. (**c**) Workflow of IgG purification and concentration from equine plasma collected by plasmapheresis. TFF - tangential flow filtration. (**d**) Analysis of purified IgG product. Polyacrylamide gel electrophoreses of purified IgG product stained with Coomassie blue (left) and analysis of purified IgG product by ELISA and virus-neutralization assays (right).

Two horses were immunized four times with 4 mg of phCMV-GP/D637L followed by a boost with 3 × 10^9^ IU of KUN-VLP-GP/D637L, with anti-GP antibody titers in horse serum samples (Fig. 1a) measured by enzyme-linked immunosorbent assay (ELISA). Low levels of anti-GP antibodies were detected in both horses after the first 2 DNA immunizations with a spike of antibodies detected at 5 weeks after the third DNA immunization (Fig. 1b). Immunization with a KUN-VLP-GP/D637L ten weeks after the fourth DNA immunization resulted in a >20 fold increase in antibody titers, which were maintained for 13 days (Fig. 1b) when plasma was collected by plasmapheresis (Fig. 1a). EBOV-neutralizing antibodies were detected only after the VLP boost, with PRNT_50_ titers of 1:160 and 1:40 produced in Horse 1 and 2, respectively (Fig. 1b, inserts). A total of 17.2 l of horse plasma was collected from both horses. IgG purification by caprylic acid precipitation according to WHO guidelines^9^ was undertaken at the BioMedical Manufacturing Fermentation Facility at CSIRO (Clayton, Australia) and produced 1.2 l of purified equine IgG with a protein concentration of 148 g/l (Fig. 1c). Gel electrophoresis and Coomassie protein staining showed ~93% purity of the final IgG product, which had an anti-GP ELISA titer of ~1:50,000 and a PRNT_50_ titer of 1:640 (Fig. 1d).

### Pilot experiment of equine anti-GP IgG administration to EBOV-infected African green monkeys

To evaluate the utility of the purified equine IgG in a post-exposure setting, two experiments were performed in NHPs. In an initial pilot experiment, four African green monkeys (*Cercopithecus aethiops*) were infected intramuscularly with 1000 PFU of the highly pathogenic Mayinga strain of Zaire EBOV^23^, and three animals were then given (by intravenous inoculation) 20 ml of equine IgG at 1 hour, 3 days and 10 days after infection. The Control untreated animal developed a rapidly escalating viremia starting on day 3, showed fever by day 5, and died on day 7 post infection (see Supplementary Fig. S1). In contrast, high level viremias in the equine IgG treated animals were delayed until day 10 after infection. The third injection of IgG on day 10 also decreased the viremias by 76 and 3.3 fold on day 11 in NHP2 and NHP3, respectively. The treatment was, however, unable ultimately to prevent the escalating viremia and subsequent mortality, with animals dying on days 13–14 (see Supplementary Fig. S1).

### Post-exposure prophylaxis using equine anti-GP IgG in Ebola infected cynomolgus macaques

**Figure 2:**
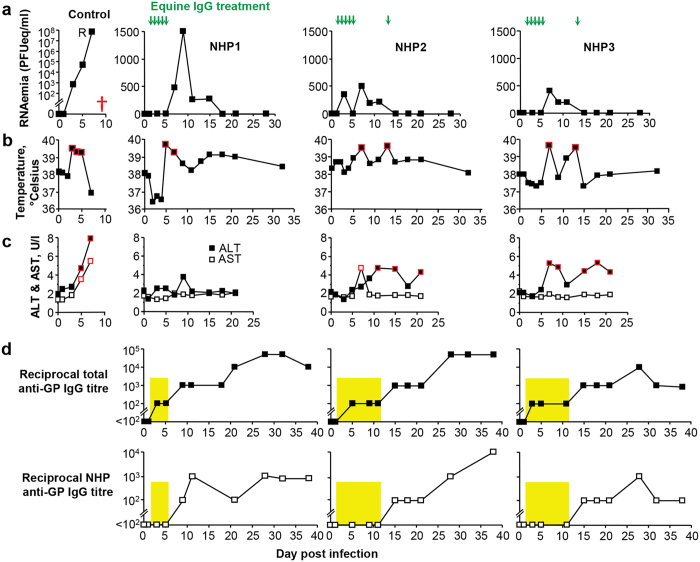
Post-exposure treatment of EBOV-infected NHPs with purified anti-EBOV GP equine IgG. Cynomolgus macaques (*Macaca fascicularis*) were infected with 1000 PFU of EBOV. The first equine IgG treatment (20 ml intravenously) was given 24 h post-infection (day 1) followed by daily treatments for 4 days (20 ml intravenously), with NHP2 and NHP3 receiving an additional treatment (5 ml intravenously) on day 13 post-infection. (**a**) EBOV RNA (RNAemia) levels in the Control and IgG-treated NHPs expressed in PFU equivalents per ml of serum (see Materials and Methods). Red cross in the Control shows time of death. (**b**) Body temperature of Control and IgG-treated NHPs. Red boxes represent ≥1.5 °C fever. (**c**) ALT and AST levels in Control and IgG-treated NHPs. Red boxes represent values above the normal range. (**d**) Analysis of anti-GP antibody responses by ELISA assays; total anti-GP antibody titers (top panels) and NHP anti-GP titers (bottom panels). Yellow boxes illustrate periods when equine GP-specific IgG, but not NHP GP-specific IgG, were detected.

Encouraged by the significant delays in mortality and reductions in viremia seen in the pilot, a second experiment was undertaken with more intensive treatment. In addition, cynomolgus macaques (*Macaca fascicularis*) were chosen as they better reflect the course of disease in humans and are widely used for evaluating the protective efficacy of anti-EBOV antibodies^4^,^10^,^24^,^25^,^26^. Four animals were infected with 1000 PFU of EBOV (Mayinga strain). The Control animal developed a rapidly escalating viremia (reaching ~7 × 10^7^ PFUeq/ml on day 7) (Fig. 2a, Control), >1.5 °C of fever (with temperatures >39.5 °C) on days 3–5 (Fig. 2b, Control, red squares), substantially elevated ALT and AST levels (Fig. 2c, Control, red squares), and died on day 9 (Fig. 2a, Control). The Control animal also developed a moderate rash on day 7 with 20–25% of the skin affected in the armpit, torso, and groin areas. Twenty four hours after EBOV infection, equine IgG treatments were initiated in three animals (NHP1, NHP2, and NHP3), with animals receiving once daily treatments of 20 ml of purified IgG for 5 days (Fig. 2a, green arrows). NHP2 and NHP3 received an additional treatment with 5 ml of purified IgG on day 13, when these animal developed a second spike of >1.5 °C of fever (Fig. 2b, red squares). All three treated animals showed a low viremia (≤1500 PFUeq/ml), >5 logs lower than the Control on day 7, with the viremias becoming undetectable on days 15–18 (Fig. 2a). All three animals developed >1.5 °C of fever on or after day 5 (Fig. 2b, red squares), and NHP2 and NHP3 developed significantly elevated ALT and/or AST levels (Fig. 2c, red squares); however, all animals survived. Anti-EBOV antibody levels were monitored in the treated animals using (i) a Protein A ELISA, which measures total EBOV-specific antibody responses (both equine and NHP) (Fig. 2d, top panels) and (ii) an ELISA measuring only NHP-specific anti-EBOV IgG responses, which represent endogenous antibody responses raised by the NHPs in response to infection (Fig. 2d, bottom panels). All treated NHPs developed their own anti-EBOV immune responses as illustrated by anti-GP IgG responses, evident 9–15 days post-infection (Fig. 2d). The yellow panels highlight the periods where equine EBOV-specific, but no NHP EBOV-specific, antibody responses were detected (Fig. 2d). Thus equine IgG administration provided serum anti-GP antibody titers in the NHPs of ~1:100 for up to 11 days post infection.

## Discussion

Herein we show 100% protection of NHPs against lethal EBOV exposure using treatment with purified equine IgG, generated by immunizing horses with a GP-DNA prime, KUN replicon-GP VLP boost, protocol. The KUN-VLP boost substantially increased both EBOV-specific IgG and neutralizing titers, with KUN virus known naturally to infect horses^27^. Although the ability of the treatment substantially to suppress the viral load is likely central to efficacy, the fall in circulating EBOV levels to undetectable levels coincided with generation by the NHPs of their own immune responses to EBOV. Serum levels of NHP anti-GP IgG were evident within 9–15 days post-infection, a similar time frame to that seen in human EBOV survivors, in whom such antibody responses are seen 8–10 days post onset of disease^28^. As the serum half-life of equine IgG in humans is about 3–4 days^29^, immune responses (both antibody and T cell^30^) raised by the NHPs to EBOV may be important for ultimately suppressing EBOV infection after the equine IgG levels have waned. The suppression of virus replication by the equine IgG may thus provide the animals with sufficient time to develop their own protective immune responses^31^. Such responses may also protect against future exposures to EBOV^32^. Treatment intensity may have been insufficient in the pilot experiment (Fig. S1), allowing EBOV to emerge before the NHPs’ own protective immune responses had adequately developed.

Potential complications arising from the use of equine IgG such as anaphylaxis and serum sickness^33^, are ameliorated (although not abolished^34^) through the use of monoclonal antibodies. However, modern protocols for purification and manufacture (recommended by WHO) have lead to substantial improvements in the safety profile of equine IgG products^9^. There is a consensus that appropriately purified immunoglobulins are very rarely the cause of adverse events more serious than a mild discomfort to a minority of patients^35^. Currently equine IgG products are both manufactured and widely used in several low income countries primarily for envenomations, with, for instance, Saharan Africa producing 377,500 vials in 2010/2011, equating to approximately 83,000 complete treatments^36^.

During an Ebola outbreak anti-EBOV equine IgG might primarily find utility as a post exposure prophylactic for individuals identified as having a high risk exposure. The sophistication of the contact tracing methodology developed towards the end of the Ebola outbreak^37^ meant that at least 50% of confirmed Ebola cases were identified as registered contacts before they had symptoms (see Supplementary Table S1). Such contact tracing methodology was also implemented in the ring vaccination strategy, where contacts and contacts of contacts were vaccinated^38^. Evidence-based risk stratification of contacts now also permits individuals with a high risk of developing Ebola disease to be identified^39^,^40^, with such individuals likely to be the primary candidates for prophylactic treatment. A period of 24 hours post-infection was allowed to elapse before initiation of IgG treatment; a period also used in one group of monkeys in ZMab studies^24^. In many cases, especially healthcare workers with a known exposure, this would present sufficient time for risk assessment, safe transportation and treatment initiation. Intravenous administration of IgG would be feasible in Ebola treatment centers given that the current mainstay of Ebola treatment is intravenous fluid replacement. As the period between infection and disease can be longer in humans (average 8–10 days, range 2–21 days^41^) than in the NHP model (usually 3–5 days), IgG prophylaxis may also retain efficacy if initiated in asymptomatic contacts beyond the 24 hour period. Natural variation in Zaire EBOV strains also appears to be limited^42^, suggesting a single IgG product might be efficacious against multiple strains.

The procedures described herein provide a scalable platform for producing a low cost treatment for use in humans potentially exposed to EBOV and raises our preparedness for future outbreaks of this devastating disease.

## Methods

### Animal ethics statements

Horse experiments were approved by the Department of Agriculture and Fisheries Animal Ethics Committee (Ref. CA 2014/11/825) and complied with the “Australian code for the care and use of animals for scientific purposes” (Animal Care and Protection Act 2001).

All non-human primate (NHP) studies were performed in BSL4 facilities at the State Research Centre for Virology and Biotechnology (SRC VB) Vector and were approved by the SRC VB Vector’s Committee on Bioethics in strict compliance with the nation-wide accepted guidelines for preclinical drug studies. The primates were housed individually in stainless-steel squeeze back cages in a climate-controlled room, 21 ± 1 °C, and relative humidity 55 ± 5% with a 12 h light/dark cycle. The animals were fed daily, *ad libitum*, with specialized food and fruits. Appropriate veterinary care was provided by a qualified veterinarian. Animals were sedated using intramuscular injection of 0.05 mg/kg of Rometar (Bioveta, Czech Republic) and 0.05 mg/kg of Zoletil (Vibrac Santé Animale, France).

### Preparation of phCMV-GP/D637L DNA

The plasmid expressing D637L mutant of the RNA edited (containing 8As at editing site) GP gene of Zaire EBOV strain Mayinga under the control of a modified human cytomegalovirus promoter was constructed from phCMVGP^21^,^43^ by mutating amino acid codon 637 in EBOV GP from D to L. The plasmid DNA for immunizations was purified from transformed *E. coli* DH5α strain using EndoFree Plasmid Giga Kit (Qiagen). DNA concentrations were determined using ND-1000 NanoDrop Spectrophotometer (Biolab).

### Preparation of KUN-VLP-GP/D637L VLPs and determination of infectious titers

KUN-VLP-GP/D637L were prepared as described previously^20^ and concentrated by centrifugation through Amicon Ultra centrifugal filter devices, with a 100,000 molecular weight cut off (Millipore, Billerica, Massachusetts). Concentrated VLPs were re-suspended in Dulbecco’s modified Eagle’s medium (DMEM), and VLP titers in infectious units per ml (IU/ml) were determined by immunofluorescence assays of Vero cells infected with 10-fold serial dilutions of VLP Cells were fixed 2 days after VLP infection and stained with anti-EBOV GP monoclonal antibody (FE37, Abcam)^20^. KUN replicon RNA encoding D637L mutant of GP is substantially less cytopathic than the one encoding wild type GP, which allows production of higher KUN VLP titres in packaging cells^19^.

### Immunization of horses

An eleven year old bay Thoroughbred mare weighting 450 kg (Horse 1) and a 10 year old black Standardbred mare weighting 400 kg (Horse 2) were leased (with full husbandry, nutrition, and veterinary support) from Plasvacc Pty. Ltd. (Kalbar, Qld., Australia). All procedures were undertaken by a registered veterinarian. Horses were immunized 4 times with 4 mg of phCMV-GP/D637L plasmid DNA (0.5 mg/ml in 150 mM sodium phosphate buffer pH = 7.0) intramuscularly into 4 sites (1 ml per site), 2 sites on each side of the neck and 2 sites on each side on the rump. KUN-VLP-GP/D637L^19^,^20^ (3 × 10^9^ IU VLPs/horse in 8 ml) were injected subcutaneously into 4 sites on the neck (2 ml per site). The schedule of immunizations and blood collections is shown in Fig. 1.

### Determination of anti-EBOV GP antibody levels in horse serum samples by ELISA

Horse blood samples were collected from the jugular vein using 20 gauge needle and 12 ml vacutainer. A soluble recombinant GP of Zaire EBOV Mayinga strain incorporating amino acids 1–311 and 462–650 and a carboxyl-terminal trimerisation domain was produced in Chinese Hamster Ovary cells and purified by immunoaffinity chromatography with the Kz52 monoclonal antibody^44^. ELISA 96-well plates were prepared by coating 50 μl per well of purified recombinant EBOV GP (concentration of 2 μg/ml) in 0.05 M carbonate-bicarbonate buffer (pH 9.6) at 4 °C overnight. Antibody titers were determined by performing 2-fold serial dilution of horse serum, starting at 1:50. ELISAs were performed as described previously^45^.

### Determination of EBOV-neutralizing antibody titers in horse serum samples

Neutralization assays were performed by making 2-fold dilutions of antisera, starting at 1 in 10 dilution. Equal volumes of sera were then mixed with 100 plaque forming units (PFU) of EBOV (Mayinga strain) and incubated at 37 °C for 30 minutes. After incubation, the antisera/virus mix was overlaid onto a confluent monolayer of Vero E6 cells in a 12 well plate and allowed to adsorb for 30 minutes at 37 °C with occasionally rocking. After 30 minutes the antisera/virus mix was removed and the cells overlaid with a DMEM/1% methycellulose and the plates incubated for 7 days. The cells were then fixed with formalin for 48 hours and infected cells visualized by staining using rabbit anti-EBOV NP antibodies as described previously^46^. Fluorescent plaques were counted and plaque reduction (50% and 80% reductions) calculated.

### Plasma collection and equine IgG purification

About 11 and 6 liters of plasma were collected from Horse 1 and Horse 2, respectively, on day 13 after VLP immunization using plasmapheresis (Haemonetics Australia Pty. Ltd.; PCS2). Jugular catheters were placed using sterile technique on both sides of the horse. One side was attached to a line containing replacement Lactated Ringers (Baxter) and the other was attached to the plasmapheresis machine. The collection process was performed by drawing whole blood from the donor horse, immediately mixing it with anti-coagulant (3.2% sodium citrate stock, Haemonetics), followed by separation of plasma from cells by centrifugation in the plasmapheresis machine. Plasma was collected into EVA bags (Baldwin, Melbourne, Australia) and blood cells returned to the donor horse. For animal welfare reasons a maximum of 10% of the blood volume can be taken and plasmapheresis is terminated if the (continuously monitored) plasma protein level in the animal drops below 55 g/l. The plasma was immediately refrigerated and remained in storage at 2–8 °C until IgG purification by caprylic acid precipitation^9^. The combined 17.2 liters of plasma was aseptically transferred to single-use Cellbag (GE Healthcare, CB0010L-02) mounted onto a WAVE 25 rocker unit (GE Healthcare) with a setting of 35 RPM and 12° angle, to provide adequate mixing. When temperature reached 22–25 °C, glacial acetic acid (Sigma Aldrich, 27225) was added to adjust the pH to 5.8. After adjusting the pH, caprylic acid (Sigma Aldrich, C2875) was slowly added to a concentration of 5% (v/v) over a 5–10 minutes time period. The plasma solution was mixed for 2 hours to complete the precipitation of unwanted proteins and lipids, and was followed by centrifuged using 1 liter centrifuge pots at 7000 rpm for 10 minutes. The supernatant was carefully removed from the centrifuge pots and filtered using a coarse (2–5 μm) 3M Encapsulated filter and final 0.2 μm filter (3M Purification LifeASSURE, PDA020C01AAG1). The clarified supernatant was concentrated using a Pall tangential flow filtration (TFF) system with a 30 kDa membrane (Sartorius Stedim, 3051465951E-SG) and diafiltered using 0.9% NaCl to achieve a final protein concentration in the range of 130–160 g/l. The protein concentration was estimated by measuring absorbance using Nanodrop Lite (Thermo Fisher Scientific). The concentrated solution was sterile filtered using a 0.2 μm 3M LifeASSURE filter and stored for 23 days at 4 °C. Post storage the material was sterile filtered again and aliquoted into 25 ml glass vials. A total of 52 vials were filled, with 20 ml in each vial.

### Determination of EBOV titers for challenge

EBOV virus (strain Mayinga) titers (PFU) were determined plaque assay. Serial 10-fold dilutions were prepared in Eagle’s MEM and used to infect Vero cell monolayers in six-well plates. The cells with virus inoculum were incubated for 1 hour at 37 °C/5% CO_2_ with rocking every 15 minutes, then virus removed and cells overlayed with 2 ml of overlay medium containing Eagle MEM, 5% FBS, and 0.5% agarose. Plates were then incubated for 7 days at 37 °C/5% CO_2_. After incubation, 2 ml of an overlay medium contacting 2% neutral red stain was added to each well. After an additional 24 hours of incubation at 37 °C/5% CO_2_, the plaques were counted, and titers were calculated using the Reed and Muench method.

### Nonhuman primates (NHPs) and EBOV challenge

For the pilot experiment African green monkeys (*Cercopithecus aethiops*) 3–4 years of age with an average weight between 2.5 and 4 kg were used. For the second treatment experiment cynomolgus macaques (*Macaca fascicularis*) 6–9 years of age with an average weight between 5 and 6 kg were used. Sedated animals were challenged by intramuscular injection of 1000 PFU of Zaire EBOV, Mayinga strain.

### Treatment of EBOV-infected NHPs with equine IgG

NHPs were sedated and treated intravenously with 20 ml of purified anti-EBOV GP equine IgG delivered slowly by gravity-driven micro-drip infusion over a 20 min period. Immediately prior to each equine IgG treatment, NHPs were injected intramuscularly with 0.5 ml of prednisolone solution (30 mg/ml) as a precaution against serum sickness^47^. In the second treatment experiment NHP2 and NHP3 were additionally injected on day 13 after infection with 5 ml of equine IgG intravenously by syringe.

### Measurement of EBOV RNA levels in primate serum by qRT-PCR

NHP were sedated and bled from the saphenous vein of the foot (alternating left and right) using 21 gauge needle and 2 ml syringe. Quantitative real-time RT-PCR (qRT-PCR) was used for the measurement of EBOV RNA in plasma samples from NHPs. Samples from the NHPs were inactivated with Trizol-LS (Invitrogen; 10296–028) before removal from BSL-4, and viral RNA was purified using the Qiagen Viral RNA Mini Kit as per manufacturer’s directions. qRT-PCR using AmpliSens® EBOV Zaire-FL Kit was performed on the RotorGene 6000 (Qiagen). A standard curve for calculating viral RNA load in PFU equivalents/ml was generated by plotting EBOV titers of a reference EBOV Mayinga strain against Ct values for viral RNA detected in the corresponding 10-fold dilutions of virus. Data from the genome quantification and plaque assays were analyzed using Prism (GraphPad Software) and CompuSyn (combosyn.com).

### Measurement of anti-EBOV antibodies in primate serum by ELISA

Serum specimens were collected and analyzed for total anti-EBOV IgG titres (horse plus monkey) and monkey-specific anti-EBOV IgG titres, using an ELISA with inactivated EBOV as antigen and Protein A-HRP (total IgG) and rabbit anti-monkey IgG-HRP (monkey IgG, Sigma Cat A2054) conjugates, respectively. End point titers were determined as the highest dilution showing an OD450 that was statistically different (mean ± 3 SD) from that of serum samples from the corresponding animals taken prior to infection.

## Additional Information

**How to cite this article**: Pyankov, O. V. *et al*. Successful post-exposure prophylaxis of Ebola infected non-human primates using Ebola glycoprotein-specific equine IgG. *Sci. Rep.* **7**, 41537; doi: 10.1038/srep41537 (2017).

**Publisher's note:** Springer Nature remains neutral with regard to jurisdictional claims in published maps and institutional affiliations.

## Supplementary Material

Supplementary Information
