# Supplementary material for: Successful post-exposure prophylaxis of Ebola infected non-human primates using Ebola glycoprotein-specific equine IgG
Source: Sci Rep. 2017 Feb 3;7:41537. doi: 10.1038/srep41537 (PMC5290740; doi:10.1038/srep41537)
Supplement: Supplementary Information [file srep41537-s1.pdf]

Title: Successful post-exposure prophylaxis of Ebola infected non-human primates using Ebola glycoprotein-specific equine IgG.

Authors: Oleg V Pyankov,[1*] Yin Xiang Setoh,[2*] Sergey A Bodnev,[1] Judith H Edmonds,[2] Olga G Pyankova,[1] Stepan A Pyankov,[1] Gabor Pali,[2] Shane Belford,[3] Louis Lu,[4] Mylinh La,[4] George Lovrecz,[4] Valentina A Volchkova[e], Keith J Chappell,[2] Daniel Watterson,[2] Glenn Marsh,[6] Paul R Young,[2] Alexander A Agafonov,[1] Jillann F Farmer,[7] Victor E Volchkov,[5] Andreas Suhrbier,[8#] Alexander A Khromykh.[2#]

Affiliations:

[1]State Center for Virology and Biotechnology Vector, Koltsovo, Russian Federation.

[2]Australian Infectious Diseases Research Centre, School of Chemistry and Molecular Biosciences, University of Queensland, St Lucia, Brisbane, QLD, Australia.

[3]Plasvacc Pty. Ltd., Kalbar, QLD, Australia.

[4] Bio Medical Manufacturing, Fermentation and Protein Production Facility, CSIRO, Clayton, VIC, Australia.

[5]Molecular Basis of Viral Pathogenicity, CIRI, INSERM, U1111-CNRS UMR5308, Université de Lyon, Université Claude Bernard Lyon 1, Ecole Normale Supérieure de Lyon, France.

[6]Australian Animal Health Laboratory, CSIRO Health and Biosecurity, Geelong, VIC, Australia

[7]United Nations Medical Service, New York, NY 10017, USA.

[8]QIMR Berghofer Medical Research Institute, Brisbane, QLD, Australia.

Supplementary data

Supplementary Fig 1

Supplementary Table 1

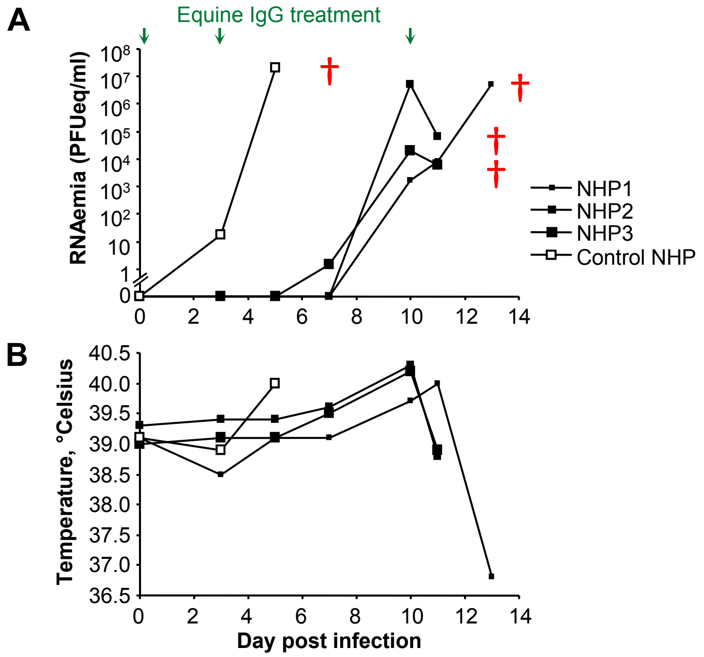

Fig. S1. Pilot experiment; therapeutic treatment of EBOV-infected African green monkeys with purified anti-EBOV GP equine IgG. Animals were infected with 1000 PFU of EBOV. The first equine IgG treatment (20 ml intravenously) was given 1 hour post-infection (day 0) followed by 2 additional treatments (20 ml intravenously) on days 3 and 10 post-infection. (**A**) EBOV RNA (RNAemia) levels in control and IgG-treated NHPs. RNAemia is expressed in PFU equivalents per ml of serum (see Materials and Methods). Red crosses show the time of death. (**B**) Body temperatures of control and IgG-treated NHPs.

WHO Ebola situation reports	Guinea			Sierra Leone		
Date	Number of new cases	Number of new cases from registered contacts	% of new cases from registered contacts	Number of new cases	Number of new cases from registered contacts	% of new cases from registered contacts
4 November 2015	1	1	100%	0	--	--
28 October 2015	3	3	100%	0	--	--
21 October 2015	3	1	33%	0	--	--
14 October 2015	0	0	--	0	--	--
7 October 2015	0	0	--	0	--	--
30 September 2015	4	4	100%	0	--	--
23 September 2015	2	0	0%	0	--	--
16 September 2015	0	0	N/A	5	4	80%
9 September 2015	1	1	100%	1	1	100%
2 September 2015	2	1	50%	1	0	0%
19 August 2015	3	3	100%	0	--	--
12 August 2015	2	1	50%	1	0	0%
5 August 2015	1	1	100%	1	1	100%
29 July 2015	4	4	100%	3	2	67%
22 July 2015	22	21	95%	4	2	50%
15 July 2015	13	9	69%	14	11	79%
8 July 2015	18	12	67%	9	5	56%
1 July 2015	12	10	83%	8	4	50%
24 June 2015	12	6	50%	8	4	50%
17 June 2015	10	5	50%	14	13	93%
10 June 2015	16	11	69%	15	8	80%
3 June 2015	13	9	69%	12	7	58%
27 May 2015	9	0	78%	3	2	67%
20 May 2015	27	5	19%	8	4	50%
13 May 2015	7	1	14%	2	2	100%
6 May 2015	9	4	44%	9	2	22%
29 April 2015	22	6	27%	11	5	50%
22 April 2015	21	10	46%	12	5	44%
15 April 2015	28	12	44%	9	6	67%
8 April 2015	21	10	48%	9	5	56%
1 April 2015	57	30	53%	25	17	67%
25 March 2015	45	16	36%	33	28	84%
18 March 2015	95	27	28%	55	37	67%
**TOTAL**	**483**	**224**	**46%**	**272**	**175**	**64%**

Supplemental Table 1. Using WHO Ebola situation reports 18th March till 4th November 2015 (a subset of reports for which complete data was available), the number of new confirmed Ebola cases and the number of these that had previously been identified by contact tracing (registered contacts) is shown. Combining data from both countries 53% of new Ebola cases occurred within registered contacts. The epidemic had largely abated in Liberia during this time period. The reports were obtained from http://who.int/csr/disease/ebola/situation-reports/archive/en/.
